# The Role of Direct Oral Anticoagulants in Cancer-Associated Thrombosis According to the Current Literature

**DOI:** 10.3390/medicina57090960

**Published:** 2021-09-12

**Authors:** Petroula Nana, Konstantinos Dakis, Michail Peroulis, Nikos Rousas, Konstantinos Spanos, George Kouvelos, Eleni Arnaoutoglou, Miltos Matsagkas

**Affiliations:** 1 Vascular Surgery Department, Larissa University Hospital, Faculty of Medicine, School of Health Sciences, University of Thessaly, 41110 Larissa, Greece; kostasdakis1994@gmail.com (K.D.); rousasnik@gmail.com (N.R.); spanos.kon@gmail.com (K.S.); geokouv@gmail.com (G.K.); milmats@gmail.com (M.M.); 2 Unit of Minimally Invasive Vascular Surgery, Mediterraneο Hospital, 16675 Athens, Greece; mperoulis@gmail.com; 3 Anesthesiology Department, Larissa University Hospital, Faculty of Medicine, School of Health Sciences, University of Thessaly, 41110 Larissa, Greece; earnaout@gmail.com

**Keywords:** cancer-associated thrombosis, venous thromboembolism, direct oral anticoagulants, recommendations

## Abstract

Venous thromboembolism (VTE) is a common complication among patients suffering from malignancies, leading to an increased mortality rate. Novel randomized trials have added valuable information regarding cancer-associated thrombosis (CAT) management using direct oral anticoagulants (DOACs). The aim of this study is to present an overview of the current literature and recommendations in CAT treatment. A few randomized control trials (RCTs) have been integrated suggesting that DOACs may be effectively applied in CAT patients compared to low molecular weight heparins (LMWHs) with a decreased mortality and VTE recurrence rate. However, the risk of bleeding is higher, especially in patients with gastrointestinal malignancies. Real-world data are in accordance with these RCT findings, while in the currently available recommendations, DOACs are suggested as a reliable alternative to LMWH during the initial, long-term, and extended phase of treatment. Data retrieved from the current literature, including RCTs and “real-world” studies, aim to clarify the role of DOACs in CAT management, by highlighting their benefits and remarking upon the potential adverse outcomes. Current recommendations suggest the use of DOACs in well-selected patients with an increasing level of evidence through years.

## 1. Introduction

Venous thromboembolism (VTE) is a common complication among patients suffering from malignancies, while the risk of VTE varies during the progression of the disease [1]. Different mechanisms, risk factors, and biological indicators have been related to cancer-associated thrombosis (CAT) and could be differentiated into patient, tumor, and treatment related [2,3]. Regarding patients suffering from CAT, the risk of fatal pulmonary embolism as well as fatal bleeding is expected to be significantly higher compared to that among the general population [4]. Thus, CAT patients represent a special group concurrently presenting a high risk for life-threatening thrombotic and bleeding events. Chemotherapy may be an additional significant VTE-associated mortality risk factor [5]. The estimated incidence of VTE in patients under chemotherapy is 9%, while the associated VTE mortality presents a 47-fold increase compared to that among the overall population [5].

In 2016, the revised American Community of Chest Physicians (ACCP) guidelines recommended the use of low molecular weight heparin (LMWH) over vitamin K antagonists (VKA) or direct oral anticoagulants (DOACs) in patients suffering from CAT [6]. In recent years, novel studies and randomized controlled trials (RCTs) have added further information regarding CAT management, and newer data have been included in the more recent recommendations provided by many specialized societies. The aim of this study is to present the current literature and recommendations regarding the role of DOACs in CAT management.

## 2. RCTs Comparing DOACs to Standard Treatment (LMWH/VKA) in CAT

In 2014, a pooled analysis of two RCTs, (Oral Direct factor Xa inhibitor Rivaroxaban in Patients with Acute Symptomatic Deep-Vein Thrombosis or Pulmonary Embolism, EINSTEIN-DVT and EINSTEIN-PE trials,) compared rivaroxaban to enoxaparin and VKAs in CAT patients [7]. In terms of VTE recurrence, both therapeutic options offered similar results in terms of efficacy (5% vs. 7%, respectively; hazard ratio (HR) 0.67, 95% confidence interval (CI) 0.35 to 1.30), while the risk of major bleeding was significantly lower in the arm of rivaroxaban (HR 0.42, 95% CI 0.18 to 0.99). The analysis concluded that rivaroxaban presented similar efficacy and better safety compared to enoxaparin and VKAs. Analogous results were recorded in the AMPLIFY trial, which compared apixaban to standard treatment. VTE and VTE-related death rates were lower in the apixaban group (HR 0.3, 95% CI 0.11 to 0.82, *p* = *0*.07) [8]. Major bleeding events were also decreased in patients treated with apixaban (HR 0.46, 95% CI 0.09 to 2.44), without achieving, though, any statistical significance (*p* = *0*.83) [8].

In 2018, a novel pilot study (Anticoagulation Therapy in Selected Cancer patients at Risk of Recurrence of Venous Thromboembolism, SELECT-D trial) which compared rivaroxaban to dalteparin in CAT patients was published [9]. Patients with hematologic and solid cancer were not excluded from the analysis [9]. The 6-month cumulative VTE recurrence rate was lower in the rivaroxaban group (4% vs. 11%, HR, 0.43; 95% CI, 0.19 to 0.99), while the 6-month cumulative rate of major bleeding was increased in the rivaroxaban group (6% vs. 4%, HR, 1.83; 95% CI, 0.68 to 4.96) [9]. It is of note that the intracranial and fatal bleeding events were equal between groups, while most major bleeding events were associated with gastrointestinal malignancies [9]. In the overall study population, the difference between hemorrhagic events was estimated at 2.4% in favor of rivaroxaban. When patients suffering from gastrointestinal malignancies were excluded, the estimated difference in bleeding events was 0.9% [9]. The 12-month follow-up of the same study confirmed the encouraging initial results with a 4% VTE recurrence rate (vs. 14%) and no major bleeding events in the rivaroxaban group [10]. The SELECT-D trial was underpowered to detect a strong statistically significant reduction in recurrent VTE with extended anticoagulation.

Similar results were confirmed in the Hokusai-VTE-Cancer trial. A lower recurrent VTE event rate and a higher bleeding event rate were recorded in the edoxaban group compared to dalteparin [11,12,13]. Most hemorrhagic events were associated with gastrointestinal malignancies. Specifically, the recurrent VTE rate was 11.3% in the dalteparin group vs. 7.9% in the edoxaban group (HR 0.71, 95% CI 0.48 to 1.06), while the major bleeding risk was estimated at 4.0% in the LMWH group vs. 6.9% in the edoxaban group (HR 1.77, 95% CI 1.03 to 3.04). Despite the higher bleeding risk in patients treated with edoxaban, bleeding events representing a clinical emergency, such as those with hemodynamic instability or intracranial hemorrhages with neurologic symptoms, were equal between treatment groups (2.3% (12/524) in the dalteparin group vs. 2.3% (12/522) in the edoxaban group).

The apixaban and dalteparin in active malignancy associated venous thrombosis (ADAM) trial, which compared the efficacy and safety of apixaban to dalteparin in CAT by randomizing a total of 300 patients, demonstrated a decreased recurrent VTE rate with the use of DOAC compared to LMWH (0.7% in the apixaban group vs. 6.3% in the dalteparin group, *p* = 0.0281). No statistically significant difference was achieved in terms of major bleeding between groups (0% vs. 1.4% in the apixaban and dalteparin groups, respectively, *p* = *0*.138) [14]. The latest available data on the use of apixaban in CAT patients arise from the Apixaban versus Dalteparin for the Treatment of Acute Venous Thromboembolism in Patients with Cancer (CARAVAGGIO) study, a prospective randomized, open label, blind endpoint evaluation, non-inferiority clinical trial. This trial also assessed the efficacy and safety of apixaban compared to dalteparin for the treatment of VTE in cancer patients [15,16]. In total, 1168 patients were enrolled and randomized between groups. The primary outcome was VTE recurrence, including DVT of the lower or upper limbs and pulmonary embolism at 6 months. The study concluded that apixaban was noninferior to subcutaneous dalteparin for the treatment of CAT (7.9% vs. 5.6% in the dalteparin and apixaban group, respectively), without increasing the 6-month major bleeding risk [15,16,17].

It is of note that the CARAVAGGIO trial included a smaller proportion of patients with upper gastrointestinal cancer and hematologic malignancies compared to other studies (4% in the apixaban and 5.4% in the dalteparin group) [15,16]. Notably, the same study excluded patients with primary and/or metastatic brain lesions, and thrombocytopenia (platelet count <75.000/μL; compared to the 50.000 platelets/μL threshold used in the SELECT-D and Hokusai trials) [9,11,15]. A meta-analysis that assessed the recurrent VTE and bleeding rates of these RCTs concluded that DOACs are associated with a nonsignificant lower risk of recurrent events and higher bleeding risk [18]. Given the heterogeneity of the available trials, it is inappropriate to conclude that DOACs are more beneficial in CAT management [19,20]. DOAC selection should rely on a detailed patient-specific approach including the type and stage of cancer, patient history, bleeding risk, and concomitant medications.

## 3. Real-World Data on CAT Patients

The Xa inhibition with Rivaroxaban for Long-term and Initial anticoagulation in venous thromboembolism (XALIA) study was a multicenter, prospective, non-interventional study which assessed the safety and effectiveness of rivaroxaban compared to standard anticoagulation in DVT patients for at least 3 months [21]. The type, dose, and duration of anticoagulation for each patient were at the physician’s discretion [21]. This study published a subgroup analysis regarding the management of patients suffering from CAT in 2017 [22]. In patients with CAT treated with rivaroxaban, the VTE recurrence, mortality, and bleeding rates were lower compared to standard treatment, even though most CAT patients were managed using LMWHs.

These outcomes were also confirmed by the US claims database analysis, which concluded that patients with newly diagnosed cancer, treated with rivaroxaban for VTE, presented a lower recurrent VTE rate compared to LMWHs, while the bleeding rate was comparable between groups during the initial 6 months [23,24]. Both apixaban and rivaroxaban, when used in daily clinical practice, presented encouraging results in terms of VTE recurrence and mortality compared to LMWHs [25,26]. However, the bleeding event rate was higher in the DOAC group [25,26].

These results were enforced by additional studies supporting the increased patient-reported treatment satisfaction with DOACs compared to standard anticoagulation [18,27,28,29]. The COSIMO trial assessed the patient-reported treatment satisfaction according to the ACTS Burden score at 4 weeks after conversion from standard treatment to rivaroxaban. The analysis concluded that patients with CAT that received rivaroxaban were more satisfied than patients under standard treatment (*p* < 0.0001) [27].

## 4. Current Recommendations on CAT Management

As the experience in DOACs’ application expands, their use increases in the daily clinical practice and includes different patient populations. In 2018, the International Society of Thrombosis and Hemostasis (ISTH) guidance recommended the use of DOACs, including only edoxaban and rivaroxaban, in cancer patients with acute VTE, low bleeding risk, and no other pharmaceutical contraindication [30]. Patients suffering from gastrointestinal cancer, with or without mucosal abnormalities, were excluded due to the associated higher bleeding risk [30]. Specifically, the ISTH guidelines separated the CAT population into two groups according to the primary malignancy-related bleeding risk. Patients with low bleeding risk were driven to be treated with rivaroxaban or edoxaban, while in the remaining population, LMWHs were preferred [30].

A year later, the American Society of Clinical Oncology (ASCO) guidelines recommended that CAT patients with acute VTE could be managed with LMWH, unfractionated heparin (UFH), fondaparinux, or rivaroxaban [31]. Except the UFH, the remaining pharmaceutical factors were proposed for long-term management, while the recommendation was similar for patients needing extended anticoagulation [31]. In 2019, the European Society of Cardiology recommendations suggested the use of edoxaban as an alternative to LMWH in gastrointestinal cancer patients (Class IIa, Level of evidence B), while rivaroxaban should be considered in patients without gastrointestinal malignancies (Class IIa, Level of evidence C) [32,33]. The difference on the level of evidence was based on the available to the literature trials’ design [32]. An analogous strategy was also suggested by the International Initiative on Thrombosis and Cancer (ITAC) guidelines regarding acute, long-term, and extended anticoagulation in CAT [34].

The latest recommendations in CAT were published in 2021 by the American Society of Hematology (ASH) and European Society for Vascular Surgery (ESVS) [35,36]. In the ASH recommendations, the use of DOACs is recommended as thromboprophylaxis in ambulatory patients with cancer and high thrombotic risk due to systemic therapy [35]. The recommendation includes only rivaroxaban and apixaban and relied on a moderate level of evidence, while the application of DOACs is not proposed in low or moderate VTE risk ambulatory patients [35]. DOACs (including apixaban, edoxaban, and rivaroxaban) are suggested as an alternative to LMWH during the initial phase (7 days), as standard treatment for three to six months, and as an option for long-term treatment of more than six months [35].

The ESVS, using a meta-analytic approach of the currently available RCTs, suggested LMWHs as the standard of treatment in CAT, while a switch to DOACs is recommended only for the extended phase in high thrombotic risk patients [36]. As previously mentioned, the group of gastrointestinal and genitourinary malignancy patients are considered as high hemorrhagic risk [36]. In the remaining CAT cases and with a careful patient selection, an approved DOAC may be used as an initial, long-term, and extended treatment (Class IIa, Level of Evidence A) [36].

Despite the differences recorded between the strategies suggested by the societies, it should be acknowledged that DOACs seem to find their role as thromboprophylaxis and VTE treatment in CAT patients (Table 1). However, the comparison of the currently available recommendations is hampered by the different approach used by the societies for the conduct of recommendations and systems applied to report the level of evidence (Table 1). It is of note that through these recent years, the level of evidence tends to rise, as more data are available regarding the role of anticoagulants in CAT management.

## 5. Considerations on the Current Literature

The main clinical question is when to choose an LMWH, VKA, or DOAC in patients suffering from CAT. The factors that mainly affect this decision are cancer, VTE, and patient related and should be taken under consideration in each patient separately. In terms of cancer-related factors, LMWHs are suggested over DOACs in patients suffering from gastrointestinal or genitourinary malignancies, while patients with solid tumors could be managed with DOACs [36,37]. Furthermore, the phase of the disease affects the physician’s decision. Patients with active or metastatic malignancies may be treated using LMWHs, while patients at cancer remission may be managed safely with DOACs. Especially when considering patients with upper gastrointestinal metastatic cancer, DOACs, which present a comparable efficacy, are associated with a higher bleeding risk [32,33,38]. Regarding VTE-related factors, in the initial phase, a preference over LMWH is justified without excluding DOAC, application while in the principal (3–6 months) and extended phase (>6 months), DOACS seem to be beneficial compared to standard treatment [32,33]. However, when an extended use of anticoagulation is needed, a half-dose policy may also be considered. A special group is represented by patients under chemotherapy or suffering from gastrointestinal disorders. This group may benefit from LMWH, while ambulatory or steady disease patients may be preferably managed with DOACs [35]. Regarding VKAs’ use in CAT, DOACs and LMWHs seem to be more effective in VTE management, with reduced or equal bleeding risk compared to VKAs [38,39]. However, it is of note that all three treatment options were equal regarding all-cause mortality [38]. Patients’ preference over an oral anticoagulant than an injectable LMWH may affect the final clinical decision [40].

Both treatment options, i.e., LMWHs and DOACs, are reliable solutions in VTE cancer-associated management and expand the spectrum of pharmaceutical choices in CAT patients [39]. A switching between these therapeutic options might be necessary during the disease course, while it seems to be safe and easy to manage in the daily clinical practice. The high thrombotic and bleeding risk in CAT patients, as in other groups of patients at excessive risk for adverse events, suggests that an individualized approach and treatment adjustment is crucial for a safe management and increased patient compliance [37,41,42]. Bleeding risk assessment is of major importance, as the related morbidity and mortality are high, and should be assessed during patients’ surveillance [42].

## 6. Conclusions

Data retrieved from the current literature, including RCTs and “real-world” studies, aimed to clarify the role of DOACs in CAT management, by highlighting their benefits and remarking upon the potential adverse outcomes. Current recommendations suggest the use of DOACs in well-selected patients with an increasing level of evidence through the years.

## Figures and Tables

**Table 1:** Current recommendations suggest direct oral anticoagulants (DOACs) as a safe and effective alternative in patients with CAT. While LMWHs are preferred in the initial phase, DOACs’ application is expanded in the principal and extended treatment phase in high thrombotic risk patients with an associated low bleeding risk.

**Societies’ recommendations**	**International Society of Thrombosis and Hemostasis (ISTH) 2018**	**In Itiative on Thrombosis and Cancer (ITAC/CME) 2019**	**European Society of Cardiology (ESC) 2019**	**American Society of Clinical Oncology (ASCO) 2020**	**American Society of Hematology (ASH) 2021**	**European Society for Vascular Surgery (ESVS) 2021**
Anticoagulant choice	Use of specific DOACS (edoxaban, rivaroxaban) and LMWHs are the preferred pharmaceutical choices (Weak guidance) LMWHs are preferred in patients with high bleeding or DDI risk (weak guidance)	Initial phase (5–10 days): LMWH, rivaroxaban, or edoxaban following ≥5 days of parenteral anticoagulation (Grade 1B) Long-term (<6 months): LMWH or DOACs (edoxaban, rivaroxaban) (Grade1A) Extended therapy (>6 months): LMWH or DOACs (Grade 1A0	In patients with PE and cancer, LMWH should be considered for the first 6 months over VKAs. (Class IIa Level A) Rivaroxaban and edoxaban should be considered as alternatives to LMWHs in patients without gastrointestinal cancer (Class IIa Level C and Class IIa Level B, respectively)	Initial phase (5–10 days): LMWH, fondaparinux or rivaroxaban preferred (evidence quality: high; strength of recommendation: strong) Long-term (<6 months): LMWH, edoxaban or rivaroxaban (VKAs are acceptable alternatives for long-term therapy if LMWH/DOACs not available) (Evidence quality: High; Strength of recommendation: strong) Extended therapy (≥6 months): LMWH, edoxaban or rivaroxaban or VKAs (evidence quality: low; strength of recommendation: weak to moderate)	DOACs (rivaroxaban, apixaban) as prophylaxis in ambulatory high thrombotic risk cancer patients under systemic therapy (moderate evidence) Initial phase (<7 days): LMWHs or DOACs (apixaban, edoxaban, rivaroxaban) as alternative (very low evidence) Long-term (3–6 months): DOACS over LMWH (Low evidence) Extended therapy (>6 months): DOACS or LMWHs (very low evidence)	LMWHs as standard of treatment in initial and principal phase (Class I Level A) Extended therapy (>6 months): DOACs (Class I Level C) DOACs as an alternative in patients without GI or genitourinary cancer for initial, principal, and extended treatment (Class IIa Level A)
**Societies’ recommendations**	**ISTH 2018**	**ITAC/CME 2019**	**ESC 2019**	**ASCO 2020**	**ASH 2021**	**ESVS 2021**
Duration of therapy	NR	LMWHs or DOACs should be used for a least 6 months, while extension should rely on individualized evaluation (Grade 1A)	Extended anticoagulation (>6 months) should be considered for an indefinite period or until cancer is cured (Class IIa Level B)	Extended therapy may be considered in active cancer (evidence quality: low; strength of recommendation: weak to moderate)	Extended anticoagulation (>6 months) should be considered for an indefinite period in active cancer (low evidence)	Extended anticoagulation (>6 months) should be considered for an indefinite period in active cancer (in text)
**Societies’ recommendations**	**ISTH 2018**	**ITAC/CME 2019**	**ESC 2019**	**ASCO 2020**	**ASH 2021**	**ESVS 2021**
Aim & weighting the evidence	To outline expert experience and the biological rational that may affect clinical decision The guidance statements are in accordance with the following premises: 1. Average patient with cancer and VTE 2. “we recommend” reflects a strong guidance with strong consensus among the panel 3. ”We suggest” reflects a weak guidance with moderate consensus among the panel	To establish a global consensus for the treatment and prophylaxis of VTE in patients with cancer The GRADE approach was used by an expert panel to conduct a systematic review of the current literature. The level of evidence was characterized as high (A), moderate (B), low (C), and very low (D), while the level of recommendation was strong (grade 1), weak (grade 2), and characterized as best clinical practice (guidance)	To suggest optimal objectively validated management strategies for patients with suspected or confirmed PE. Conclusions based on the available scientific evidence, using the European Society of Cardiology grading system (A, B, or C indicates the level of current evidence). Depending on the strength of recommendation, each one is categorized as Class I, IIa/IIb, and III.	To provide updated recommendations about prophylaxis and treatment of VTE in patients with cancer A systematic review of RCTs reporting on VTE prophylaxis and treatment using PubMed and CENTRAL databases, executed by an expert commit using the “signals” approach	To support patients, clinicians, and others in decisions about treatment of VTE The Grading of Recommendations Assessments, Development and Evaluation (GRADE) approach was used by an expert panel	To assist clinicians in selecting the best management strategies to achieve optimal patient outcomes Revision and summary of the relevant peer reviewed published literature. Conclusions based on the available scientific evidence, using the European Society of Cardiology grading system (A, B, or C indicates the level of current evidence). Depending on the strength of recommendation, each one is categorized as Class I, IIa/IIb, and III.

Footnotes: CAT: cancer-associated thrombosis; DOACs: direct oral anticoagulants.
